# Supplementary material for: Neutrophil gelatinase-associated lipocalin partly reflects the dynamic changes of renal function among chronic hepatitis C patients receiving direct-acting antivirals
Source: PLoS One. 2021 Aug 26;16(8):e0256505. doi: 10.1371/journal.pone.0256505 (PMC8389462; doi:10.1371/journal.pone.0256505)
Supplement: S1 Table — (DOCX) [file pone.0256505.s001.docx]

**Supplement table 1. Baseline characteristics of chronic hepatitis C patients receiving DAA with or without grade 2/3 renal function deterioration at P12 in nonSOF users.**

| **Variable** | | **All patients**  **(n = 120)** | **With grade 2/3 deterioration**  **(n = 29) (26.7%)** | **Without grade 2/3 deterioration**  **(n = 91) (73.3%)** | **P value** |
| --- | --- | --- | --- | --- | --- |
| **Baseline clinical characteristics** | | | | | |
| Age (years)† | | 64.06 ± 10.22 | 67.15 ± 6.30 | 63.10 ± 11.03 | 0.043 |
| Male (%) | | 41 (34.2%) | 14 (48.3%) | 27 (29.7%) | 0.076 |
| Fatty liver | | 38 (31.7%) | 15 (51.7%) | 23 (25.3%) | 0.011 |
| Hyperlipidemia | | 12 (10.0%) | 7 (24.1%) | 5 (5.5%) | 0.008 |
| Diabetes mellitus | | 26 (21.7%) | 9 (31.0%) | 17 (18.7%) | 0.196 |
| Hypertension | | 26 (21.7%) | 9 (31.0%) | 17 (18.7%) | 0.196 |
| eGFR ranks* | | | | | 0.428 |
|  | rank 1 | 38 (31.7%) | 7 (24.1%) | 31 (34.1%) |  |
|  | rank 2 | 70 (58.3%) | 19 (65.5%) | 51 (56.0%) |  |
|  | rank 3 | 12 (10.0%) | 3 (10.3%) | 9 (9.9%) |  |
| **Baseline characteristics of HCV and liver-related conditions** | | | | | |
| Advanced fibrosis (%) | | 70 (58.3%) | 19 (65.5%) | 51 (56.0%) | 0.396 |
| HCC history (%) | | 20 (16.7%) | 6 (20.7%) | 14 (15.4%) | 0.569 |
| Splenomegaly (%) | | 37 (30.8%) | 13 (44.8%) | 24 (26.4%) | 0.069 |
| Ascites (%) | | 1 (0.8%) | 0 (0.0%) | 1 (1.1 %) | 1.000 |
| Baseline HCV viral load (IU/mL)† | | 6.10Log ± 0.86Log | 5.86Log ± 1.20Log | 6.17Log ± 0.71Log | 0.512 |
| HCV genotype 1 (%) | | 110 (91.7%) | 26 (89.7%) | 84 (92.3%) | 0.703 |
| **Baseline medications associated with renal function** | | | | | |
| ACEI/ARB users | | 12 (10.0%) | 4 (13.8%) | 8 (8.8%) | 0.481 |
| Diuretics users | | 2 (1.7%) | 0 (0.0%) | 2 (2.2%) | 1.000 |
| NSAID users | | 21 (17.5%) | 5 (17.2%) | 16 (17.6%) | 1.000 |
| **Baseline laboratory data** | | | | | |
| Baseline NGAL (ng/ml) † | | 17.55 ± 9.55 | 20.59 ± 10.32 | 16.58 ± 9.14 | 0.042 |
| ALT(U/L)† | | 83.63 ± 72.30 | 99.21 ± 99.57 | 78.66 ± 61.02 | 0.498 |
| AST(U/L)† | | 60.55 ± 62.40 | 73.79 ± 86.03 | 56.33 ± 52.65 | 0.406 |
| Albumin (g/dl) † | | 4.27 ± 0.30 | 4.22 ± 0.29 | 4.29 ± 0.30 | 0.222 |
| Total bilirubin (mg/dl) † | | 0.73 ± 0.30 | 0.78 ± 0.32 | 0.71 ± 0.29 | 0.399 |
| eGFR (ml/min/1.73m^2^)† | | 80.66 ± 15.93 | 78.72 ± 13.99 | 81.28 ± 16.52 | 0.274 |
| Hb† (gm/dL) | | 13.67 ± 1.48 | 13.71 ± 1.54 | 13.65 ± 1.46 | 0.688 |
| Prothrombin time (INR)† | | 1.02 ± 0.05 | 1.04 ± 0.06 | 1.01 ± 0.05 | 0.078 |

e-GFR, estimated glomerular filtration rate; HCC, hepatocellular carcinoma; HCV, hepatitis C virus; ACEI, angiotensin-converting enzyme inhibitor; ARB, angiotensin receptor blocker; NSAID, nonsteroidal anti-inflammatory drugs; NGAL, neutrophil gelatinase-associated lipocalin; ALT, alanine aminotransferase; AST, aspartate aminotransferase; INR, international normalized ratio

*rank 1: > 90 ml/min/1.73 m2, rank 2: 60-90 ml/min/1.73 m2, rank 3: 30-60 ml/min/1.73 m2; †Data are expressed as mean±SD.
