# Supplementary material for: Neutrophil gelatinase-associated lipocalin partly reflects the dynamic changes of renal function among chronic hepatitis C patients receiving direct-acting antivirals
Source: PLoS One. 2021 Aug 26;16(8):e0256505. doi: 10.1371/journal.pone.0256505 (PMC8389462; doi:10.1371/journal.pone.0256505)
Supplement: S2 Table — (DOCX) [file pone.0256505.s002.docx]

**Supplement table 2. Baseline characteristics of chronic hepatitis C patients receiving DAA with or without grade 2/3 renal function deterioration at P12 in SOF users.**

| **Variable** | | **All patients**  **(n = 112)** | **With grade 2/3 deterioration**  **(n = 33) (26.7%)** | **Without grade 2/3 deterioration**  **(n = 79) (73.3%)** | **P value** |
| --- | --- | --- | --- | --- | --- |
| **Baseline clinical characteristics** | | | | | |
| Age (years)† | | 63.98 ± 11.14 | 65.82 ± 10.09 | 63.21 ± 11.52 | 0.255 |
| Male (%) | | 42 (37.5%) | 14 (42.4%) | 28 (35.4%) | 0.525 |
| Fatty liver | | 37 (33.0%) | 8 (24.2%) | 29 (36.7%) | 0.271 |
| Hyperlipidemia | | 6 (5.4%) | 1 (3.0%) | 5 (6.3%) | 0.668 |
| Diabetes mellitus | | 18 (16.1%) | 5 (15.2%) | 13 (16.5%) | 1.000 |
| Hypertension | | 24 (21.4%) | 7 (21.2%) | 17 (21.5%) | 1.000 |
| eGFR ranks* | | | | | 0.382 |
|  | rank 1 | 38 (33.9%) | 10 (30.3%) | 28 (35.4%) |  |
|  | rank 2 | 50 (44.6%) | 14 (42.4%) | 36 (45.6%) |  |
|  | rank 3 | 24 (21.4%) | 9 (27.3%) | 15 (19.0%) |  |
| **Baseline characteristics of HCV and liver-related conditions** | | | | | |
| Advanced fibrosis (%) | | 83 (74.1%) | 26 (78.8%) | 57 (72.2%) | 0.637 |
| HCC history (%) | | 17 (15.2%) | 8 (24.2%) | 9 (11.4%) | 0.146 |
| Splenomegaly (%) | | 34 (30.4%) | 12 (36.4%) | 22 (27.8%) | 0.377 |
| Ascites (%) | | 4 (3.6%) | 2 (6.1%) | 2 (2.5 %) | 0.580 |
| Baseline HCV viral load (IU/mL)† | | 5.88Log ± 1.08Log | 5.87Log ± 1.10Log | 5.88Log ± 1.08Log | 0.924 |
| HCV genotype 1 (%) | | 44 (39.3%) | 17 (51.5%) | 27 (34.2%) | 0.095 |
| **Baseline medications associated with renal function** | | | | | |
| ACEI/ARB users | | 13 (11.6%) | 7 (21.2%) | 6 (7.6%) | 0.054 |
| Diuretics users | | 9 (8.0%) | 4 (12.1%) | 5 (6.3%) | 0.445 |
| NSAID users | | 13 (11.6%) | 2 (6.1%) | 11 (13.9%) | 0.339 |
| **Baseline laboratory data** | | | | | |
| Baseline NGAL (ng/ml) † | | 14.54 ± 8.20 | 15.73 ± 9.27 | 14.05 ± 7.72 | 0.612 |
| ALT(U/L)† | | 89.80 ± 78.38 | 87.70 ± 63.70 | 90.67 ± 84.11 | 0.532 |
| AST(U/L)† | | 62.93 ± 46.57 | 66.06 ± 45.85 | 61.62 ± 47.10 | 0.491 |
| Albumin (g/dl) † | | 4.12 ± 0.41 | 4.07 ± 0.45 | 4.14 ± 0.39 | 0.650 |
| Total bilirubin (mg/dl) † | | 0.85 ± 0.48 | 0.87 ± 0.44 | 0.84 ± 0.50 | 0.559 |
| eGFR (ml/min/1.73m^2^)† | | 77.10 ± 19.54 | 74.41 ± 19.31 | 78.22 ± 19.65 | 0.330 |
| Hb† (gm/dL) | | 13.32 ± 1.75 | 13.28 ± 1.52 | 13.34 ± 1.85 | 0.959 |
| Prothrombin time (INR)† | | 1.04 ± 0.08 | 1.05 ± 0.08 | 1.04 ± 0.08 | 0.529 |

e-GFR, estimated glomerular filtration rate; HCC, hepatocellular carcinoma; HCV, hepatitis C virus; ACEI, angiotensin-converting enzyme inhibitor; ARB, angiotensin receptor blocker; NSAID, nonsteroidal anti-inflammatory drugs; NGAL, neutrophil gelatinase-associated lipocalin; ALT, alanine aminotransferase; AST, aspartate aminotransferase; INR, international normalized ratio

*rank 1: > 90 ml/min/1.73 m2, rank 2: 60-90 ml/min/1.73 m2, rank 3: 30-60 ml/min/1.73 m2; †Data are expressed as mean±SD.
