# Supplementary material for: Neutrophil gelatinase-associated lipocalin partly reflects the dynamic changes of renal function among chronic hepatitis C patients receiving direct-acting antivirals
Source: PLoS One. 2021 Aug 26;16(8):e0256505. doi: 10.1371/journal.pone.0256505 (PMC8389462; doi:10.1371/journal.pone.0256505)
Supplement: S3 Table — (DOCX) [file pone.0256505.s003.docx]

**Supplement table 3. Patient factors associated with grade 2/3 renal function deterioration in chronic hepatitis C patients receiving DAA at P12^ for nonSOF and SOF-based DAA users.**

|  | **Odds Ratio (95% CI)** | **P value** |
| --- | --- | --- |
| **NonSOF-based DAA users** | | |
| Sex  Female  Male | 1.000  3.161 (1.150–8.686) | 0.026 |
| Fatty liver  No  Yes | 1.000  4.684 (1.689–12.990) | 0.003 |
| Hyperlipidemia  No  Yes | 1.000  9.401 (2.268–38.959) | 0.002 |
| BL NGAL | 1.051 (1.000–1.104) | 0.05 |
| **SOF-based DAA users** | | |
| ACEI/ARB users  No  Yes | 1.000  3.276 (1.008–10.647) | 0.049 |

^Adjusted for age, sex, variables with P < 0.1 from supplement table 1: fatty liver, hyperlipidemia, splenomegaly, BL NGAL and PT INR and from supplement table 2: GT1 and ACEI/ARB users for nonSOF users and SOF users, respectively ; factors reported to be associated with renal injury: baseline renal disease, DM, HTN, liver disease (advanced fibrosis), ACEI/ARB users, diuretics users, and NSAID users were also considered in multivariate analysis.
